# Serum Magnesium and Bone Health in Pediatric Nephrotic Syndrome: A Cross-Sectional Study From South Asia

**DOI:** 10.7759/cureus.105172

**Published:** 2026-03-13

**Authors:** Shaifalika Thakur, Sanat K Thakur, Tanwi Singh, Sunil Kishore

**Affiliations:** 1 Pediatrics, Indira Gandhi Institute of Medical Sciences, Patna, IND; 2 Pediatrics, Shri Sai Hospital, Patna, IND; 3 Pediatric Cardiology, Heart Hospital, Patna, IND; 4 Cardiology, U. N. Mehta Institute of Cardiology and Research Centre, Ahmedabad, IND; 5 Cardiology, Heart Hospital, Patna, IND; 6 Pathology, Indira Gandhi Institute Of Medical Sciences, Patna, IND; 7 Pathology, Shri Sai Hospital, Patna, IND

**Keywords:** bone metabolic disease, primary nephrotic syndrome, serum magnesium, steroid resistant nephrotic syndrome, steroid sensitive nephrotic syndrome

## Abstract

Background

Metabolic bone disease (MBD) is common in pediatric nephrotic syndrome (NS), yet magnesium (Mg)-a co-factor for vitamin D metabolism and parathyroid hormone (PTH) secretion-has been under-studied.

Objective

To evaluate serum Mg status in children with NS and its relationship with bone health and mineral metabolism.

Methods

We conducted a cross-sectional study of consecutively enrolled children with NS (N=370) at a tertiary center. Demographics, NS phenotype, treatment exposure, dual-energy X-ray absorptiometry (DEXA)-based bone density, and laboratory parameters-including Mg, calcium, phosphorus, alkaline phosphatase (ALP), 25-hydroxyvitamin D [25(OH)D], and intact PTH-were recorded. Associations between Mg and clinical/laboratory variables were assessed using t-tests/ analysis of variance* (*ANOVA), Pearson correlations, and multivariable models.

Results

A total of 370 pediatric patients with NS were included (mean age: 6.02 ± 3.3 years; 263 males, 71.1%). NS phenotypes included: steroid-resistant NS (SRNS, n=47, 12.7%), frequently relapsing NS (FRNS, n=173, 46.8%), steroid-dependent NS (SDNS, n=82, 22.2%), and infrequent relapsers NS (IFRNS, n=62, 16.8%). The mean serum magnesium (sMg) level was 1.73 ± 0.42 mg/dL (range: 0.8-2.9 mg/dL). Children with SRNS had significantly lower sMg levels compared to steroid-sensitive NS (SSNS) (1.35±0.36 vs. 1.75±0.42 mg/dL; p=0.002). No significant associations were found between sMg and demographic or clinical variables (all p>0.05). The sMg showed a negative correlation with PTH levels (r=−0.118; p=0.027) but no significant correlation with serum calcium, phosphorus, ALP, or 25(OH)D levels.

Conclusions

Lower Mg is associated with higher PTH and reduced bone density in pediatric NS, independent of high rates of vitamin D deficiency. Routine Mg assessment may help risk-stratify bone health and represents a modifiable target for future trials.

## Introduction

Nephrotic syndrome (NS), characterized by nephrotic-range proteinuria, edema, hypoalbuminemia, and hyperlipidemia, is one of the most common chronic glomerular disorders in children. It is frequently complicated by metabolic bone disease (MBD), primarily due to urinary losses of vitamin D-binding protein, prolonged glucocorticoid exposure, and reduced physical activity [1,2]. These factors collectively disrupt bone metabolism and contribute to an increased risk of impaired bone mineralization in affected children.

While the effects of calcium and vitamin D on bone health in pediatric NS have been extensively studied, magnesium (Mg)-a mineral central to parathyroid hormone (PTH) secretion and the activation of vitamin D-has received comparatively little attention [3]. This knowledge gap is noteworthy because Mg plays a critical role in bone mineralization, osteoblastic activity, and overall skeletal health [4-7].

Outside the context of NS, multiple studies in both children and adults have demonstrated that low serum magnesium (sMg) levels are associated with reduced bone mineral density (BMD) and altered PTH and vitamin D signaling [4,5,8]. Additionally, randomized clinical trials have shown that dietary Mg supplementation can positively influence bone mass accrual in children and adolescents [7,9]. In patients with NS, preliminary studies suggest that sMg levels may fluctuate with disease activity, with lower concentrations reported during active proteinuric phases compared to remission [1-3,10]. However, data linking Mg status to bone health outcomes in this population remain limited and inconsistent [6,8,11].

Given these gaps in evidence, the present study aimed to (1) evaluate sMg status in children with NS and (2) assess its association with BMD and markers of mineral metabolism, including serum calcium, phosphorus, alkaline phosphatase (ALP), vitamin D, and PTH.

## Materials and methods

This was a hospital-based cross-sectional study conducted at a single tertiary care center in India over two years (January 2022 to December 2024) to evaluate Mg status and its association with bone health in pediatric patients with NS.

Participants

Inclusion Criteria

Children aged one to 14 years diagnosed with NS based on standard clinical criteria (proteinuria ≥40 mg/m²/hour or urine protein/creatinine ratio >2, hypoalbuminemia <2.5 g/dL, and edema) were included in the study.

Exclusion Criteria

Patients with chronic kidney disease stage 3 or higher, known MBD, chronic liver disease, endocrine disorders affecting bone metabolism, or those receiving medications known to influence Mg metabolism were excluded from the study.

The final sample size was calculated using standard statistical methods and determined to be 370 patients to ensure adequate power for detecting significant associations. The required sample size was calculated using standard power analysis methods, assuming a prevalence of hypomagnesemia in NS of approximately 35-40% based on prior literature, with a confidence level of 95% and a margin of error of 5%. The minimum required sample size was estimated to be approximately 350 participants. To account for potential missing data, a total of 370 children were included in the final analysis.

Measurements

BMD was assessed using dual-energy X-ray absorptiometry (DEXA). Results were interpreted using age- and sex-matched pediatric Z-scores. Reduced bone density was defined as a BMD Z-score ≤ −2.0, in accordance with International Society for Clinical Densitometry (ISCD) pediatric guidelines. Laboratory evaluations included sMg levels (colorimetric assay; reference range 1.46-2.68 mg/dL), total calcium, phosphorus, alkaline phosphatase (ALP), 25-hydroxy vitamin D [25(OH)D], and intact PTH levels, following standardized protocols [3,6].

Variables

Patients were further classified based on their NS subtype. (1) steroid-sensitive nephrotic syndrome (SSNS), which included infrequent relapsers, frequent relapsers, and (2) steroid-dependent patients; and steroid-resistant nephrotic syndrome (SRNS). Treatment-related variables included duration and cumulative dose of corticosteroids, exposure to calcineurin inhibitors and other immunosuppressive drugs, and the use of calcium and vitamin D supplements. Additional variables included current relapse status, presence of infections, and other clinical complications documented during follow-up.

Definitions

NS phenotypes were classified according to standard pediatric nephrology criteria. SSNS was defined as remission achieved within four weeks of daily corticosteroid therapy. SRNS was defined as failure to achieve remission after four weeks of standard-dose daily prednisolone therapy. Steroid-dependent nephrotic syndrome (SDNS) was defined as two consecutive relapses occurring during steroid tapering or within 14 days of discontinuation of steroid therapy. Frequently relapsing nephrotic syndrome (FRNS) was defined as two or more relapses within six months of initial response or four or more relapses within any 12-month period.

Statistical analysis

Data were analyzed using appropriate parametric and non-parametric statistical tests. Group comparisons were performed using independent t-tests or one-way analysis of variance (ANOVA) for continuous variables and chi-square (χ²) tests for categorical variables. Pearson or Spearman correlation analyses were performed to assess associations between Mg levels, bone density, and biochemical markers. Multivariable models were built based on clinical relevance to adjust for potential confounders. Statistical significance was defined as a p-value of <0.05.

Ethical considerations

The study protocol was reviewed and approved by the Institutional Ethics Committee (Reference number: 614/IEC/IGIMS/2022; approval date: July 18, 2022). Informed consent was obtained from the parents or guardians of all participants prior to enrollment in the study, in accordance with the Declaration of Helsinki guidelines.

## Results

Cohort characteristics

A total of 370 pediatric patients with NS were included in the study. The mean age was 6.02±3.3 years, with the majority being male(n=263, 71.1%), and the gender distribution was not significantly different across NS phenotypes (χ²=2.45, degree of freedom -df=3, p=0.48, Cramér’s V=0.08).

**Figure 1:**
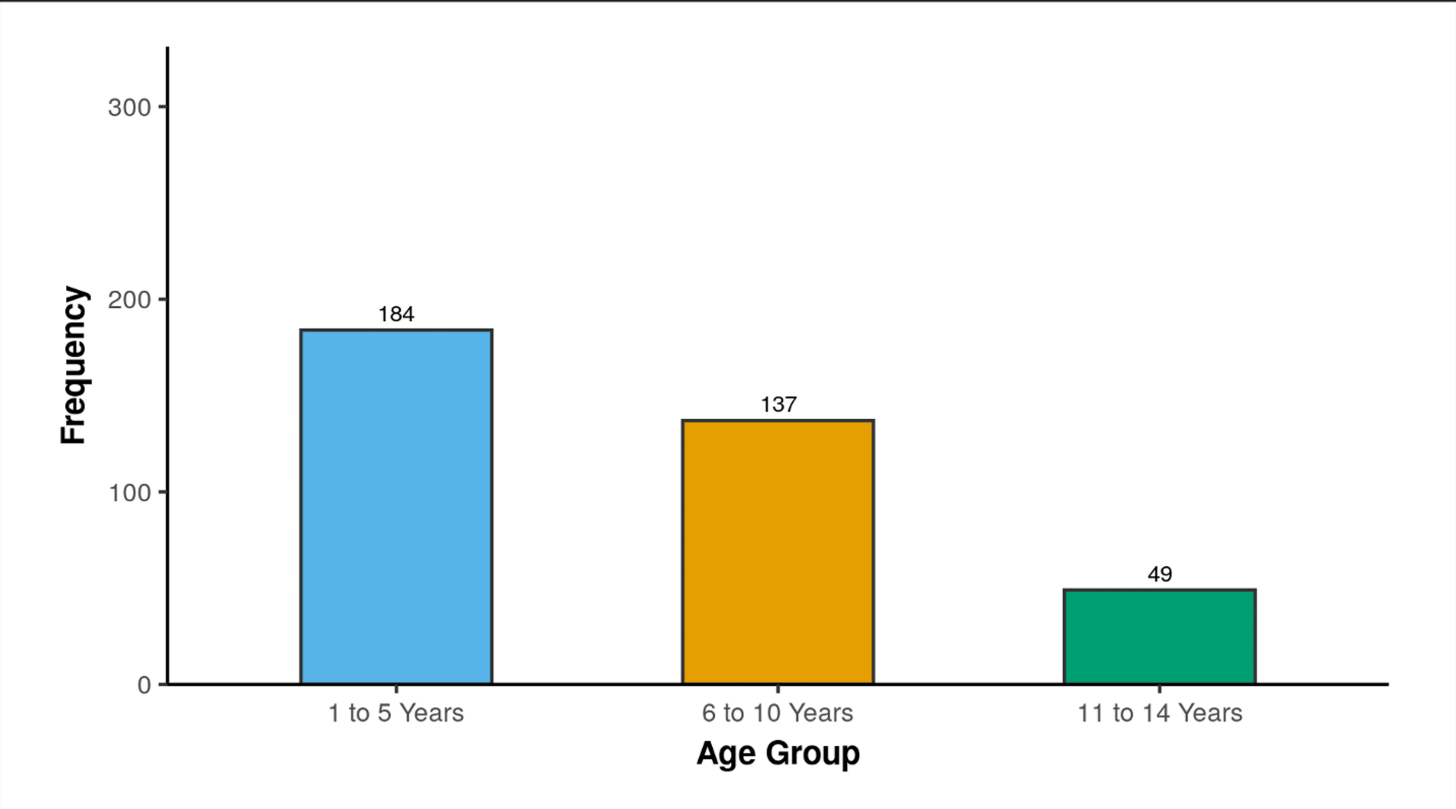
Age Distribution Of The Study Population

As seen in Figure 1, the distribution of NS phenotypes was as follows: steroid-resistant NS (SRNS) - 12.7% (n=47), frequently relapsing NS (FRNS) - 46.8%(n=173), steroid-dependent NS (SDNS) - 22.2%(n=82), and infrequent relapsers NS (IFRNS) - 16.8% (n=62).

sMg profile

Tables 1, 2 highlight that the mean sMg level in the cohort was 1.73 ± 0.42 mg/dL (range: 0.8-2.9 mg/dL). Children with SRNS(n=47, 12.7%) had significantly lower sMg levels compared to steroid-sensitive NS (SSNS, n=323, 87.3%) - (1.35 ± 0.36 vs. 1.75 ± 0.42 mg/dL; χ²=9.80, df=1, p=0.002, Cramér’s V=0.16) [1,2]. No significant associations were found between sMg and age, gender, height, weight, age at onset, or duration of illness (all p > 0.05). The sMg levels were slightly lower in children with SDNS (n=82, 22.2%) but without reaching statistical significance, as seen in Table 2.

**Table 1:** Serum Magnesium in Relation to Steroid Responsiveness

Serum Magnesium	Steroid Sensitive	Steroid Resistant	Wilcoxon-Mann-Whitney U Test (W)	p-value
Mean (SD)	1.75 (0.42)	1.35 (0.36)		
Median (IQR)	1.79 (1.4–2.07)	1.33 (1.1–1.52)	3279.000	0.002
Min – Max	0.8 – 2.9	0.8 – 1.98		

**Table 2:** Association of Clinical Parameters with Serum Magnesium ¹ p-value from Spearman correlation for continuous variables; ² p-value from Kruskal–Wallis test for disease pattern groups; ³ p-value from Mann–Whitney U test for steroid responsiveness and dependence; **- p<0.01 (highly statistically significant). Steroid responsiveness categories - SS: steroid sensitive; SR: steroid resistant; SD: steroid dependent. p<0.05 is considered statistically significant. NS: nephrotic syndrome; IFRNS: infrequent relapsers nephrotic syndrome; FRNS: frequently relapsing nephrotic syndrome.

Parameters	Serum Magnesium (Mean ± SD / Correlation Coefficient)	p-value
Age (Years)	Correlation Coefficient (rho) = 0.01	0.908¹
Age Group		0.923²
1 to 5 Years	1.74 ± 0.43	
6 to 10 Years	1.73 ± 0.43	
11 to 14 Years	1.72 ± 0.41	
Gender		0.638³
Male	1.74 ± 0.42	
Female	1.71 ± 0.42	
Weight (Kg)	Correlation Coefficient (rho) = -0.03	0.615¹
Height (cm)	Correlation Coefficient (rho) = -0.02	0.739¹
Age of Onset of NS (Years)	Correlation Coefficient (rho) = -0.02	0.687¹
Duration of Illness (Years)	Correlation Coefficient (rho) = -0.02	0.725¹
Pattern		0.130²
First Episode	1.77 ± 0.40	
First Relapse	1.77 ± 0.43	
IFRNS	1.63 ± 0.38	
FRNS	1.70 ± 0.46	
Steroid Responsiveness*		0.002³ **
Steroid Sensitive	1.75 ± 0.42	
Steroid Resistant	1.35 ± 0.36	
Steroid Dependence		0.090³
Steroid Dependent	1.66 ± 0.43	
No	1.75 ± 0.42	

Bone density and mineral metabolism

In this study, reduced bone density was observed in 14.9% (n=55) of patients with a significant association between reduced bone density and low sMg levels (χ²=6.05, df=1, p=0.014, Cramér’s V=0.13). Vitamin D deficiency (<20 ng/mL) was present in 66.8% (n=247), while hypocalcemia (<8.4 mg/dL) and elevated ALP were seen in 16.8% (n=62) and 10.5% (n=39), respectively. Children with reduced bone density(n=55, 14.9%) had significantly lower Mg levels compared to those with normal density (1.62±0.44 vs. 1.73±0.41 mg/dL; p=0.045) [4].

**Figure 2:**
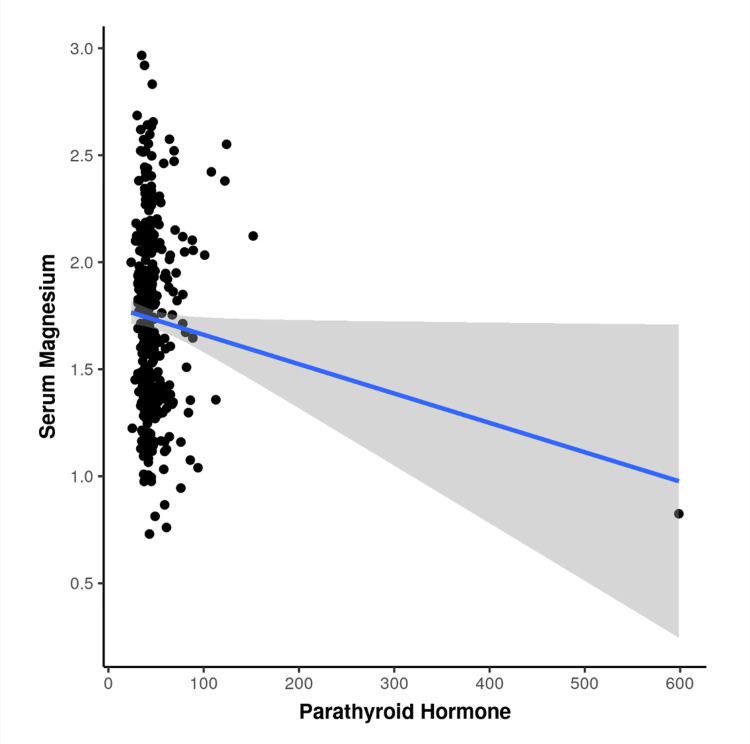
Scatter Plot Showing the Relation Between Serum Magnesium and PTH levels Serum Mg in mg/dl, Serum PTH in pg/ml

A significant and relevant finding of this study is depicted in Figure 2, which shows that sMg was negatively correlated with PTH levels (r = −0.118; p = 0.027). No significant correlations were observed with serum calcium, phosphorus, ALP, or 25(OH)D levels [3,5].

## Discussion

Principal findings

In this large pediatric cohort (N=370), we observed that lower sMg levels were associated with elevated PTH and reduced BMD. The lowest Mg concentrations were consistently seen in patients with SRNS. These findings expand on prior smaller studies by examining the full clinical spectrum of NS and linking Mg status to markers of bone health and mineral metabolism.

Comparison with prior work

Our findings are consistent with earlier reports. A Romanian study of 27 children found that sMg levels were significantly lower during active NS and fluctuated between remission and relapse phases [1]. In pediatric remission cohorts, hypomagnesemia has been reported in 36-52% of patients, particularly in those with SRNS [2]. Beyond NS, evidence in healthy children and adolescents indicates a positive correlation between sMg and BMD [4], and randomized controlled trials have shown that dietary Mg supplementation enhances hip bone mineral accrual during adolescence [7]. Similar associations between low sMg and reduced BMD have also been observed in adults [8].

Mechanistic plausibility

Mg plays a critical role in mineral metabolism and skeletal health. It modulates PTH secretion, vitamin D activation, and the Receptor Activator of Nuclear Factor-κB (RANK)/ Receptor Activator of Nuclear Factor-κB Ligand (RANKL)/ Osteoprotegerin (OPG) pathway, influencing osteoclast differentiation and bone resorption [9,12]. Experimental animal models have demonstrated that Mg deficiency can increase bone resorption, reduce osteoblast function, and impair bone formation [9].

One mechanistic explanation for the inverse correlation between sMg and PTH is skeletal PTH resistance. Chronic Mg depletion impairs PTH receptor function in bone, reducing the intracellular cyclic adenosine monophosphate (AMP) response despite elevated circulating PTH [13]. Mild hypomagnesemia may initially stimulate PTH secretion via activation of the calcium-sensing receptor (CaSR), but severe hypomagnesemia can paradoxically suppress PTH release by impairing CaSR function [14]. Additionally, an inverse correlation between Mg and PTH has also been documented in patients receiving peritoneal dialysis, supporting a suppressive effect of Mg on PTH synthesis [15].

Prolonged corticosteroid therapy, which is a cornerstone of NS management, may itself influence Mg homeostasis and bone metabolism. Corticosteroids can increase renal Mg excretion and alter intracellular Mg distribution, potentially contributing to lower sMg levels. Additionally, chronic steroid exposure has independent effects on bone metabolism, including stimulation of osteoclast activity, suppression of osteoblast function, and alterations in PTH regulation. Therefore, corticosteroid exposure may represent an additional factor contributing to the observed associations between Mg levels, PTH, and BMD in children with NS.

Clinical implications

Our findings emphasize the need to integrate Mg status into the assessment of bone health in pediatric NS. Given the high prevalence of vitamin D deficiency in these children, Mg repletion may act synergistically with calcium and vitamin D supplementation to optimize bone metabolism. This is particularly relevant for SRNS patients and those receiving calcineurin inhibitors. Pediatric studies have demonstrated that Mg supplementation enhances bone mass accrual [7,9], suggesting a potential therapeutic avenue.

Strengths and limitations

Key strengths of this study include its large sample size, DEXA-based BMD assessment, and comprehensive biochemical evaluation. However, the cross-sectional design limits causal inferences, and dietary Mg intake was not assessed. The single-center setting may also affect generalizability. Nevertheless, the consistent inverse association between sMg and PTH, even in the context of widespread vitamin D deficiency, suggests a biologically plausible and robust relationship. The sMg and calcium supplementation were not included in this study, which may have affected the results obtained.

The present study did not evaluate the effect of Mg supplementation on bone metabolism. Therefore, although an association between lower sMg levels and altered mineral metabolism was observed, causal relationships and therapeutic benefits of Mg supplementation cannot be established from this study.

Future directions

Longitudinal studies and interventional trials are warranted to determine whether Mg supplementation can improve bone health and normalize mineral metabolism in pediatric NS, particularly in SRNS and calcineurin inhibitor-treated subgroups. Future research should incorporate dietary assessments, serial DEXA evaluations, and measurements of bone turnover markers to elucidate these relationships further. Prospective longitudinal studies and randomized controlled trials are required to determine whether Mg supplementation can improve BMD and normalize mineral metabolism in children with NS.

## Conclusions

This study highlights the significant role of sMg in the mineral metabolism and bone health of children with NS, with lower Mg levels independently associated with higher PTH concentrations and reduced bone density, particularly in steroid-resistant NS, making this subgroup especially vulnerable to metabolic bone complications. Routine monitoring of serum Mg, along with calcium and vitamin D, may offer a more comprehensive assessment of bone health risk, and given the mechanistic links between Mg, PTH regulation, vitamin D activation, and bone remodeling, Mg repletion could serve as an adjunct strategy to prevent or mitigate MBD. Prospective studies and interventional trials are warranted to evaluate whether optimizing Mg status can improve bone outcomes, especially in high-risk SRNS patients and those receiving calcineurin inhibitors.
